# A cross-sectional analysis on the effects of biodiversity and green space on mental well-being in Wales.

**DOI:** 10.23889/ijpds.v7i3.1996

**Published:** 2022-08-25

**Authors:** Oliver Thwaites

**Affiliations:** 1 Swansea University

## Objectives

A growing body of evidence suggests that biodiversity and greenspaces are positively associated with mental well-being. However, studies have been undertaken over small spatial scales and population-scale evidence is lacking. This study aimed to investigate associations between parks, biodiversity and mental well-being for individuals living in Wales in 2018/2019.

## Approach

This cross-sectional study linked GIS derived data, socio-demographic and survey datasets to analyse how mental well-being varied with biodiversity per greenspace for individuals living in Wales for each Lower Super Output Area (LSOA). Mental well-being was defined using the Warwick-Edinburgh Mental Well-being Scale (WEMWBS). All records of bird, butterfly and plant species in 2018 were downloaded from the National Biodiversity Network Atlas Wales. Greenspace measures were acquired from Ordnance Survey. Associations between greenspace, biodiversity and mental well-being were investigated using Generalised Additive Models to allow for non-linear relationships.

## Results

The size of the cohort was 10,441, the average age was 55 (s.d. 18.6), 55.2% were female and 94.4% were White British. This study found that greenspace area (m2), the total number of species, number of bird and number of plant species (all per m2 greenspace) had positive statistically significant relationships with mental well-being. After adjustment for socio-demographic factors, the number of plant species (per m2 greenspace) remained a significant predictor of mental well-being, with a positive linear relationship. There was no statistically significant relationship between the number of butterfly species per m2 greenspace and mental well-being in unadjusted or adjusted models. Furthermore, socio-demographic factors explained most of the variation in mental well-being.

## Conclusion

This study adds further evidence to the benefits of biodiverse environments on mental well-being through nature connectedness. Specifically, people living in an LSOA with more plant species per m2 greenspace report better mental well-being. Environmental interventions should consider increasing plant species richness which may benefit mental well-being.

